# Advancing Hyperspectral Image Analysis with CTNet: An Approach with the Fusion of Spatial and Spectral Features

**DOI:** 10.3390/s24062016

**Published:** 2024-03-21

**Authors:** Dhirendra Prasad Yadav, Deepak Kumar, Anand Singh Jalal, Bhisham Sharma, Julian L. Webber, Abolfazl Mehbodniya

**Affiliations:** 1 Department of Computer Engineering & Applications, G.L.A. University, Mathura 281406, Uttar Pradesh, India; dhirendra.yadav@gla.ac.in (D.P.Y.); asjalal@gla.ac.in (A.S.J.); 2 Department of Computer Engineering, NIT Meghalaya, Shillong 793001, Meghalaya, India; deepak.kumar@nitm.ac.in; 3 Chitkara University Institute of Engineering and Technology, Chitkara University, Rajpura 140401, Punjab, India; 4 Department of Electronics and Communication Engineering, Kuwait College of Science and Technology (KCST), Doha Area, 7th Ring Road, Kuwait City 13133, Kuwait; j.webber@kcst.edu.kw

**Keywords:** hyperspectral, RGB, fusion, spatial, feature, classification, transformer

## Abstract

Hyperspectral image classification remains challenging despite its potential due to the high dimensionality of the data and its limited spatial resolution. To address the limited data samples and less spatial resolution issues, this research paper presents a two-scale module-based CTNet (convolutional transformer network) for the enhancement of spatial and spectral features. In the first module, a virtual RGB image is created from the HSI dataset to improve the spatial features using a pre-trained ResNeXt model trained on natural images, whereas in the second module, PCA (principal component analysis) is applied to reduce the dimensions of the HSI data. After that, spectral features are improved using an EAVT (enhanced attention-based vision transformer). The EAVT contained a multiscale enhanced attention mechanism to capture the long-range correlation of the spectral features. Furthermore, a joint module with the fusion of spatial and spectral features is designed to generate an enhanced feature vector. Through comprehensive experiments, we demonstrate the performance and superiority of the proposed approach over state-of-the-art methods. We obtained AA (average accuracy) values of 97.87%, 97.46%, 98.25%, and 84.46% on the PU, PUC, SV, and Houston13 datasets, respectively.

## 1. Introduction

Hyperspectral imaging captures highly detailed spectral information across numerous narrow bands. In contrast to traditional imaging systems that record data in only a few broad spectral channels (e.g., RGB), hyperspectral sensors can acquire data in hundreds or thousands of contiguous and narrow bands [1]. This vast amount of spectral information provides high capabilities for various applications, including agriculture, environmental monitoring, mineral exploration, urban planning, and military surveillance [2]. Hyperspectral imaging can capture the unique spectral signature of materials, surfaces, and objects. Each pixel in a hyperspectral image contains a spectral curve representing the reflectance or emissivity of the corresponding material at different wavelengths. Analyzing these spectral curves empowers researchers and practitioners to gain valuable insights into the composition and characteristics of the observed scene, enabling the identification of specific materials, vegetation species, mineral deposits, and pollution levels, among others. However, the effective utilization of hyperspectral data remains a formidable challenge. The primary obstacle arises from the high dimensionality of hyperspectral datasets, where each pixel contains a vast number of spectral bands [3]. This substantial increase in data dimensions poses difficulties for traditional image processing and classification.

Furthermore, the challenges imposed by high dimensionality and limited spatial resolution make conventional classification methods less effective. In recent years, significant progress has been made in machine learning (ML) and deep learning (DL). However, ML-based methods require handcrafted features for training. Therefore, performance could be more optimal [4]. In contrast, convolutional neural networks (CNNs) have demonstrated remarkable feature extraction, pattern recognition, and image-classification capabilities. Several CNN-based methods have utilized spatial features for land cover classification. Further, several research studies have utilized joint spectral and spatial features to improve the classification [5]. Vision transformers (ViTs) have recently been proposed to provide long-range dependency on spatial and spectral features for the classification of land objects [6].

Hyperspectral image classification categorizes pixels or regions within a hyperspectral image into predefined classes or land cover categories. Meanwhile, the supervised machine learning methods support vector machine (SVM) [7] and random forest (RF) [8] have been widely used in the early stages of hyperspectral image analysis using texture and color features of the land covers. These methods rely on spectral signatures to discriminate between different classes of land cover. However, hyperspectral data are characterized by high dimensionality, as each pixel contains many spectral bands. Researchers have thus explored various techniques to address these challenges and enhance classification accuracy. Feature-extraction methods, such as principal component analysis (PCA) [9] and minimum noise fraction (MNF) [10], have been utilized to reduce data dimensionality while preserving relevant information. Additionally, dimensionality reduction algorithms like non-negative matrix factorization (NMF) [11] and t-distributed stochastic neighbor embedding (t-SNE) [12] have been employed to enhance the separability of different classes in reduced feature spaces.

Recently, DL techniques have gained popularity in hyperspectral image classification. Deep learning models, particularly convolutional neural networks (CNNs), have demonstrated exceptional capabilities in automatically learning hierarchical and discriminative features from raw data. The CNN-based approach is extensively employed in many image-related applications because of its inherent local connectivity and translational invariance properties. In the context of HSI, CNNs are typically constructed by considering both spatial and spectral dimensions. Several studies [13] have employed a 2-D CNN approach to concurrently extract spatial and spectral information to classify hyperspectral images (HSIs). Moreover, a previous study [14] has used a three-dimensional convolutional neural network (3-D CNN) to extract spectral details for land cover classification. The study [15] proposes a novel approach called the spectral–spatial residual network (SSRN). This method combines continuous spectral and spatial residual blocks to extract feature maps from hyperspectral images. The primary objective of this approach is to mitigate the issue of gradient disappearance commonly observed in neural networks. An end-to-end residual spectral–spatial attention network (RSSAN) was suggested for hyperspectral image classification by Zhu et al. [16].

Classification accuracy is improved by combining the spatial and spectral attention modules. In their paper, Xing et al. [17] presented DIKS, a novel deep network with a self-expressive property and irregular convolutional kernels. Hyperspectral image classification was the primary motivation for developing this network. The Multilevel Feature Network and Spectral–Spatial Attention Model (MFNSAM) is a new method presented in [18]. In this approach, a CNN is integrated with the attention mechanism. A multilayer convolutional neural network (CNN) and a spectral–spatial attention module make up the MFNSAM. Fusion techniques play a pivotal role in remote sensing applications when integrating data from several sources to acquire complementary and comprehensive information about the scene under observation. Information from hyperspectral pictures is fused with data from other sources in hyperspectral image analysis, such as multispectral photos, auxiliary RGB images, or LiDAR data [19]. Improving classification accuracy, enhancing geographical features, and providing a more comprehensive picture of the scene are all goals of merging disparate data types. Hyperspectral image classification presents several unique obstacles, and various fusion methods have been investigated to solve these issues [20]. These methods include pixel-level fusion, feature-level fusion, and decision-level fusion. The spatial and spectral properties were enhanced by merging them at four scales using a lightweight deep CNN model based on residuals, as demonstrated by Li et al. [21]. In a similar study, Wang et al. [22] improved the spatial information of HSI by multispectral image through cross-modality information extracted by a multi-hierarchical cross transformer (MCT).

Pixel-level fusion involves merging individual pixels from multiple data sources to create a new image that integrates spectral and spatial information [23]. This technique is beneficial when the spatial resolution of hyperspectral images is lower than that of ancillary data sources, as it allows for enhanced spatial details in the final fused image [24]. On the other hand, feature-level fusion involves extracting features from different data sources and combining them to create a new feature representation that captures complementary information from both sources. Feature-level fusion can preserve the original data sources’ spectral and spatial characteristics while reducing data redundancy and increasing classification accuracy [25]. We have added a summary of some recent methods for hyperspectral classification in Table 1.

In short, the ML-based method fails to achieve high performance on HSI datasets due to its dependency on handcrafted features. On the other hand, the CNN approach improved performance but lacked correlation with the long-range features. Further, ViT improved the long-range dependency of the spatial and spectral features. However, computational costs also increased. In the hyperspectral image, spatial resolution is low and spectral resolution is high due to continuous narrow spectral bands. When classifying the land covers in the hyperspectral data, spatial and spectral features play crucial roles. In an RGB image, spatial resolution is high, and spectral resolution is low. Our primary motivation was to improve the spatial and spectral resolution. Therefore, we designed two module-based models, including CTNet. We first generated a synthetic RGB image from the HSI data in one module using a spectral weighting technique. We utilized a pre-trained ResNeXt model to improve the spatial features. In the second module, we first reduced the dimensions of the HSI data using PCA since the processing of many bands requires high costs and time. After that, an enhanced attention-based transformer model was utilized to improve the spectral features and provide long-range dependency. Finally, spatial and spectral features were fused to classify the land covers.

The significant contributions of the method are as follows.

1. We demonstrate the effectiveness of improving spatial features through synthetic RGB images using a pre-trained ResNeXt to classify the land covers.
2. We develop and optimize a multiscale attention module of the transformer block to provide long-range dependency of the spectral features.
3. We designed a fusion module to generate enhanced spatial and spectral features obtained through convolution and transformer modules.
4. We conducted extensive experiments to evaluate the performance of the proposed method on four benchmark datasets.

The rest of the paper is arranged as follows.

In Section 2, a description of the proposed model architecture for HIS classification is discussed. Further, in Section 3, quantitative and visual results on different datasets are illustrated. In Section 4, we discuss the results, Finally, in Section 5, we discuss the conclusion, limitations, and future scope of the proposed method.

## 2. Materials and Methods

**Figure 1:**
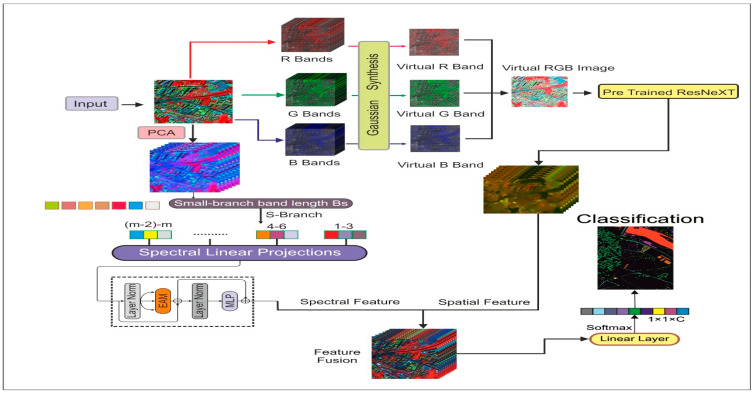
The proposed CTNet architecture for the classification of the land covers.

In the proposed study, we designed a dual-block convolution and transformer-based model. The transformer block extracts spectral features, and the convolution block enhances the spatial features using virtual RGB images. The detailed architecture of the proposed model is shown in Figure 1.

### 2.1. Enhanced Attention-Based Vision Transformer (EAVT)

Suppose the hypercube of the hyperspectral image (HSI) is $I\in R^{M\times N\times B}$, where M and N indicate the width and height dimensions, and B denotes the total number of bands. Each pixel inside image I encompasses both spatial and spectral characteristics. Their one-hot encoding is represented by a vector, denoted as $H=\left\{h_{1},h_{2},\ldots \ldots h_{C}\right\}$, where C represents the various land covers. In HSI, numerous continuous bands offer significant spectral information. However, this increased number of bands also leads to higher computational costs and redundancy. Principal component analysis (PCA) is employed on band B to address this issue. After PCA is performed, the resulting band is denoted as D, and it is represented as $Y\in R^{M\times N\times D}$. The pixel-wise spectral input is defined as $Y_{spec}=\left\{y_{1},y_{2},y_{3},\ldots \ldots y_{D}\right\}\in R^{1\times D}$. After that, the spectral band is converted to tokens, and positional encoding is performed as follows.

(1) $$Y^{\prime }=POS\left(Y_{spec}\right)=\left[Y_{CLS}||Y_{band}\right]+Y_{pos}$$

where N = number of bands in the token T, $Y_{CLS}\in R^{1\times D}$ class tokens, $Y_{band}\in R^{N\times D}$ band tokens, $Y^{\prime }$ = output after positional encoding, and $Y_{spec}\in R^{(1+N)\times D}$ is generated after position encoding.

The attention weight $A_{j}^{k}$ of the jth input with neighbor size k and relative positional bias B(i,j) is calculated as follows.

(2) $$A_{j}^{k}=\left[\begin{array}{c} \;Q_{j}K_{\sigma_{1}(j)}^{T}+B_{\left(j,\sigma_{1}(j)\right)}\\ Q_{j}K_{\sigma_{2}(j)}^{T}+B_{\left(j,\sigma_{2}(j\right)}\\ \;Q_{j}K_{\sigma_{k}(j)}^{T}+B_{\left(j,\sigma_{k}(j)\right)} \end{array}\right]$$

**Figure 2:**
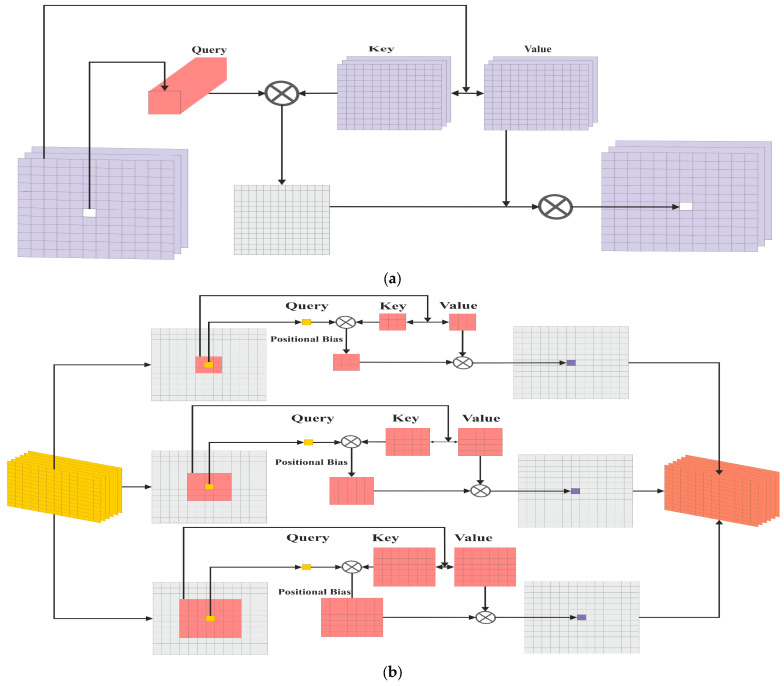
The architecture of the self-attention (**a**) and enhanced attention (**b**) block.

In Equation (1), the nearest neighbor of the *k*-th input is denoted by σ(k). Query (Q) and Key (K) are the linear projection token vectors. After that, the linear projection neighbor is calculated using Equation (2).

(3) $$V_{j}^{k}={\left[V_{\sigma_{1}(j)}^{T},V_{\sigma_{2}(j)}^{T},\ldots \ldots V_{\sigma_{j}(k)}^{T}\right]}^{T}$$

where $v_{j}^{k}$ is a matrix that represents k nearest neighbor linear projection value of the *j*-th input. Finally, the attention to the *j*-th tokens with neighbor size k is defined using Equation (4).

(4) $$T_{out}=EAT_{k}(j)=Soft\max\left(\frac{A_{j}^{k}}{\sqrt{d}}\right)V_{j}^{k}$$

where $\sqrt{d}$ is the scaling factor. The attention obtained using Equation (4) is repeated for every pixel in the feature map. The detailed architectures designed for the attention module of the classical transformer and the proposed one are shown in Figure 2.

### 2.2. Synthetic RGB Image Formation

Let H be the hyperspectral image cube with dimensions M × N × P, where M and N are the spatial dimensions (height and width) and P is the number of hyperspectral bands. We defined the intensity of the image at spatial position (I, j) in the *K*-th band with H_ijk_. Further, the weight matrix W of each RGB channel with dimension 3 × P is defined, in which the rows represent the red, green, and blue channels and the columns represent the weight for each hyperspectral band in producing the RGB channel. We applied weights to each hyperspectral band to enhance the quality and relevance of the derived data. Additionally, it optimizes computational resources for accurately identifying constituent materials and data processing for specific applications. Band weighting refines hyperspectral data, making land cover classification more accurate and efficient. We applied band weighting to each channel c and spectral band P and calculated the intensity of the spectral band as follows.

(5) $$I_{c,K}^{ij}=H_{ijk}\times W_{cK}$$

(6) $$I_{R}^{i,j}=\sum_{k=1}^{P}H_{ijk}\times W_{R,k}$$

(7) $$I_{B}^{i,j}=\sum_{k=1}^{P}H_{ijk}\times W_{B,k}$$

(8) $$I_{G}^{i,j}=\sum_{k=1}^{P}H_{ijk}\times W_{G,k}$$

where $I_{c,K}^{ij}$ = intensity of the spectral band K for channel c, at spatial position (i, j).

$H_{ijk}$ = intensity of the hyperspectral image at position (i, j) in the *k*-th band.

$W_{cK}$ = weight of the *k*-th spectral band for *c*-th RGB channel.

After this, we populated each channel for the spatial dimension at position (i, j) as follows.

(9) $$RGB\_I(i,j,1)=I_{R}^{ij}$$

(10) $$RGB\_I(i,j,2)=I_{G}^{ij}$$

(11) $$RGB\_I(i,j,3)=I_{B}^{ij}$$

For each channel (R, G, B) of the synthetic image, the minimum and maximum intensity values are calculated to ensure all channels have values within the same range. In hyperspectral images, different bands might have been captured under slightly different illumination conditions. Normalization mitigates these differences, ensuring that the brightness and contrast are consistent across bands. The normalization operation is performed as follows.

(12) $$I_{N}(i,j,1)=\frac{RGB\_I(i,j,1)-\min Val_{R}}{\max Val_{R}-\min val_{R}}\times 255$$

(13) $$I_{N}(i,j,2)=\frac{RGB\_I(i,j,2)-\min Val_{G}}{\max Val_{G}-\min val_{G}}\times 255$$

(14) $$I_{N}(i,j,3)=\frac{RGB\_I(i,j,3)-\min Val_{B}}{\max Val_{B}-\min val_{B}}\times 255$$

where $I_{N}(i,j,1)$ = normalized intensity pixel value of red channel at a spatial position (i, j). RGB_I(i,j,1) = intensity of the pixel at spatial position (i, j) in the red channel before normalization. $\min val_{R}$ = minimum intensity value of red channel. $\max val_{R}$ = maximum intensity of red channel. $I_{N}(i,j,2)$ = normalized intensity pixel value of blue channel at a spatial position (i, j). RGB_I(i,j,2) = intensity of the pixel at spatail position (i, j) in blue channel before normalization. $\min val_{R}$ = minimum intensity value of the blue channel. $\max val_{R}$ = maximum intensity of the blue channel. $I_{N}(i,j,3)$ = normalized intensity pixel value of green channel at a spatial position (i, j). RGB_I(i,j,3) = intensity of the pixel at spatail position (i, j) in the green channel before normalization. $\min val_{R}$ = minimum intensity value of green channel $\max val_{R}$ = maximum intensity of green channel.

After normalization, we rounded each pixel value to the nearest integer in each channel, and finally, the image was constructed as follows.

(15) $$I_{RGB}=[round(I_{N}(i,j,1),round(I_{N}(i,j,2),round(I_{N}(i,j,3)]$$

The synthesized RGB image is passed to the pre-trained ResNeXt for spatial feature extraction. The Algorithm 1 to generate synthetic RGB is shown below.
**Algorithm 1:** Steps to generate synthetic RGB image**Input:** Hyperspectral image cube H, with dimensions M × N × P and Weight matrix (W)(1) For each channel (Red, Green, Blue) and each spectral band (P), calculate the intensity of the spectral band as follows.                    $I_{\left(c,K\right)}^{i,j}=H_{i,j,k}\times W_{c,K}$ where $I_{\left(c,K\right)}^{i,j}$ = intensity of *k*-th spectral band for channel c at spatial position (i, j). $H_{ijk}$ = intensity of the hyperspectral image at position (i, j) in the *k*-th band. $W_{cK}$ = weight of the *k*-th spectral band for *c*-th RGB channel. (2) Calculate the intensity of the R, G, and B channels for the synthetic image using Equation (6), Equation (7) and Equation (8), respectively. (3) For each channel (R, G, B) at spatial position (i, j), populate the channel with calculated intensities using Equation (9), Equation (10) and Equation (11), respectively. (4) Normalize each pixel value in the R, G, and B channels by calculating minimum and maximum values using Equation (12), Equation (13) and Equation (14), respectively. (5) Round each pixel value to the nearest integer in each channel as follows.                   I_N=round(I_N(i,j,c)) where I_N = Normalized pixel value rounded to the nearest integer and I_N(i,j,c) = normalized intensity value of the pixel at position (i, j) in channel c. (6) Construct the final RGB image using the normalized and rounded values in each channel as follows.                   RGB_final(i,j,c)=I_N(i,j,c) where RGB_final(i,j,c) = pixel value in the final RGB image at position (i, j) in channel c and $\text{I\_N}\left(i,j,c\right)\;$= normalized and round intensity value of pixel value at position (i, j) in channel c. **Output:** RGB image

### 2.3. Enhanced Spatial Features Using Virtual RGB Images

**Figure 3:**
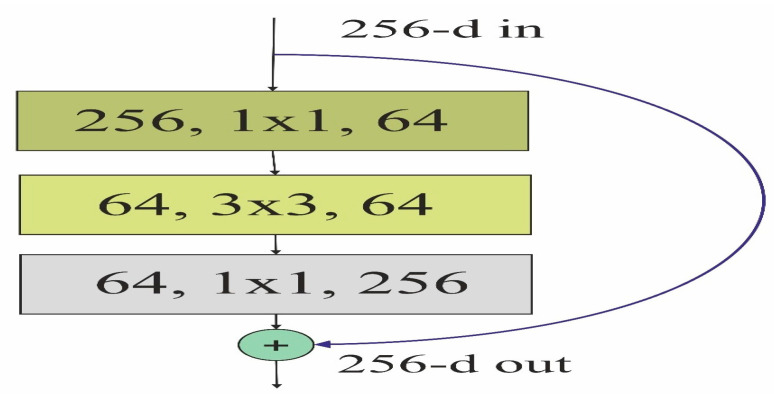
The residual block of the model.

The labeled hyperspectral image data are limited. Significant differences in imaging settings, spectral bands, and ground objects make hyperspectral data unsuitable for training using nature. The CNN can classify HSI by determining the pixel level of each land cover. In the proposed study, we utilized a three-channel synthetic RGB image to enhance spatial features using a pre-trained ResNeXt model trained on natural images for pixel-level classification. A residual block is mathematically defined as follows.

(16) $$y=F(x,\{W_{i}\})+x$$

where F represents the residual, x is the input, and y is the output. In the ResNeXt, the input is split into several branches, processing each distinctively and subsequently merging them. The split and merge function are expressed as follows for a specific layer.

(17) $$F(x)=\sum_{i=1}^{C}T(x,W_{i})$$

where C is the cardinality and T represents each branch’s transformation function. The residual block is shown in Figure 3, and the branch’s transformation is represented as follows.

**Figure 4:**
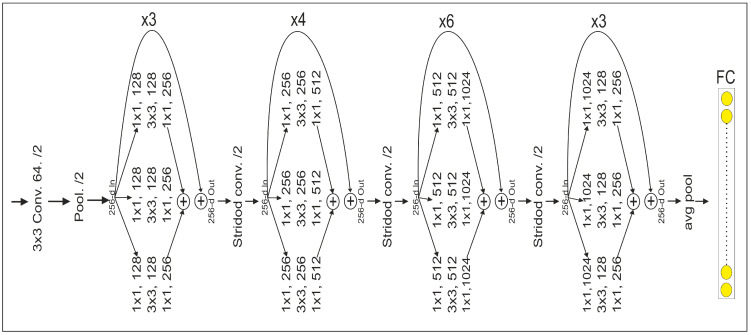
Architecture of the ResNeXt for spatial feature extraction.

(18) T(x,W)=conv(Re⁡LU(BN(conv(Re⁡LU(BN(x,W1))),W2)))

where T(x, w) is the output of the convolution layer for input x, “conv” refers to the convolutional process, “BN” indicates batch normalization, “ReLU” is the rectified linear activation, and W_1_ and W_2_ are convolutional operation weights. After that, a global average pooling and a fully connected layer are added to classify the land covers. The detailed architecture of the ResNeXt model is shown in Figure 4.

### 2.4. Spectral–Spatial Feature Fusion for HSI Classification

We observed that the spatial features obtained through FCN and spectral features extracted by transformer blocks differed in the range and distribution of values. Therefore, features were normalized and integrated to merge spatial and spectral features. Suppose $T_{out}\in R^{W\times H\times D_{s}}$ is a spectral feature obtained from a Transformer with band D_s_ and $C_{out}\in R^{W\times H\times D_{spa}}$ is a spatial feature obtained from pre-trained FCN with dimension D_spa_. Here, W×H is the size of the size of the feature that will be fused to generate an enhanced features vector. Before the fusion process, spectral features are normalized as follows.

(19) $$\begin{array}{c} \overset{\sim }{T_{d}}=\frac{1}{W\times H}\sum_{i=1}^{n}\sum_{j=1}^{n}T_{ij}\\ \sigma_{d}=\frac{1}{W\times H}\sum_{i=1}^{n}\sum_{j=1}^{n}(T_{ij}-\overset{\sim }{T}d)\\ F(T_{ij})=\left(\frac{T_{ij}-\overset{\sim }{T_{d}}}{\sigma_{d}}\right)/\left|\frac{T-\overset{\sim }{T_{d}}}{\sigma_{d}}\right| \end{array}$$

where $\overset{\sim }{T_{d}}$ = mean of the spectral features, $\sigma_{d}$ = standard deviation, $F(T_{ij})$ = normalized spectral features.

Similarly, we normalized the spatial feature. After normalization, we concatenated the spectral and spatial features to generate an enhanced feature vector as follows.

(20) $$F_{e}=concat(F_{out},C_{out})$$

Finally, the enhanced F_e_ is passed to the Softmax layer for the classification of the land covers. The loss of the model on each dataset having N training samples is calculated as follows.

(21) $$Loss=-\frac{1}{N}\sum_{j}\sum_{p=1}^{P}I_{jp}\log(v_{jp})$$

where P = Total land cover categories, I_jp_ = Indicator function. It takes a value of 1 if the *j*-th category is p and otherwise 0. V_jp_ = Probability value of the *j*-th samples belongs to *p*-th class. The Algorithm 2 for the proposed method is shown below.
**Algorithm 2:** The proposed method’s algorithm**INPUT:** Hyperspectral image $I\in R^{H\times W\times D}$ and ground truth label $X\in R^{H\times W}$.1. Apply PCA and set dimension D = 30, and pass it to the transformer block.2. Generate RGB image from $I\in R^{H\times W\times D}$ using spectral weighting.3. For I = 1 to 200, do   (a) Train the ResNeXT using synthesize image.   (b) Apply spectral linear projection to generate Q, K, and V and pass to EAVT.   (c) Train the EAVT. end4. Apply Equations (19) and (20) to generate enhanced features.5. Test the model for classification of land covers.6. Plot the training loss curve.**OUTPUT:** Classified label of the test dataset ($I\in R^{H\times W\times C}$)

## 3. Experimental Results and Discussion

In this section, we have demonstrated the quantitative and visual results obtained on four datasets.

### 3.1. Datasets Description

In this section, we discussed the datasets used to evaluate the proposed method for hyperspectral image classification. Four benchmark hyperspectral datasets, PU (Pavia University), PUC (Pavia University Centre), SV (Salina Valley), and Houston-13, were selected for this study. The PU dataset captures an area covering Pavia University, Italy, and was acquired by the Reflective Optics System Imaging Spectrometer (ROSIS) sensor. The spatial dimensions of the dataset are 610 × 340 pixels, and each pixel represents a ground area of 1.3 m × 1.3 m, which is the spatial resolution of the dataset, which has nine land cover classes. The PUC dataset has spatial dimensions of 1096 × 715 pixels. The image in PUC is larger than the Pavia University dataset, which has 610 × 340 pixels. It contains 102 bands, and it has nine land cover classes.

**Figure 5:**
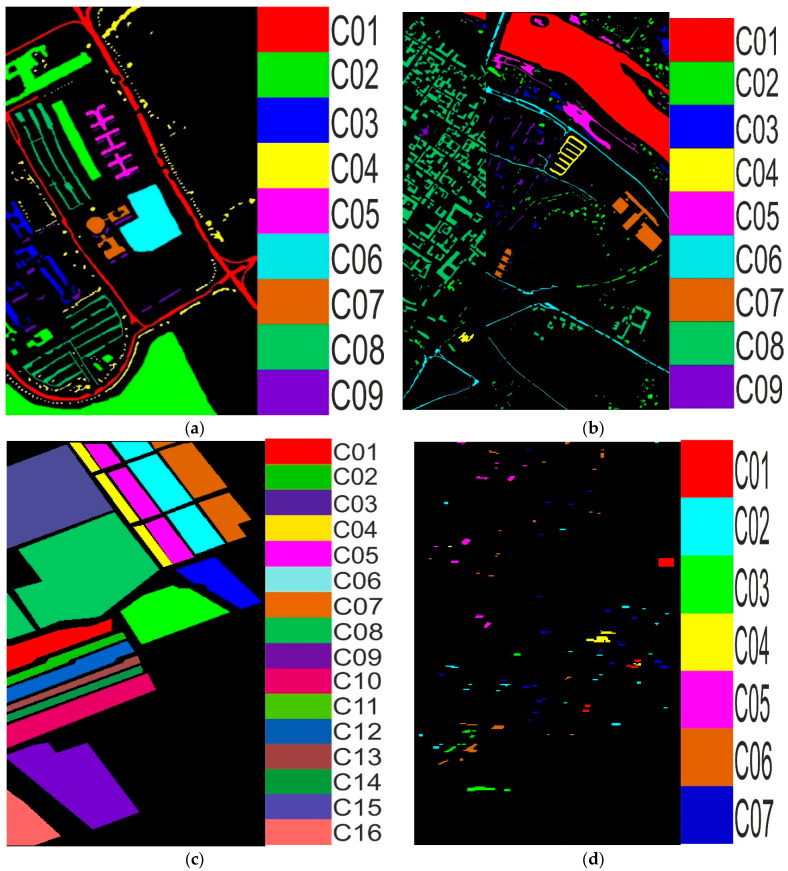
The ground truth map with the class label colors of the PU, PUC, SV, and Houston13 shown in (**a**), (**b**), (**c**), and (**d**), respectively.

The SV data were captured using the Airborne Visible/Infrared Imaging Spectrometer (AVIRIS) sensor, and they have spatial dimensions of around 512 × 217 pixels. The dataset typically contains 224 contiguous spectral bands. The spectral bands usually cover a range from 0.4 µm to 2.5 µm, and they have 16 land covers. The Houston13 dataset contains both hyperspectral and LiDAR data from an urban area in Houston, Texas, USA. This dataset has 144 spectral bands in the 380 nm to 1050 nm region and has been calibrated to at-sensor spectral radiance units. The spatial dimensions of the dataset are 349 × 1905 pixels, with a spatial resolution of 2.5 m. The detailed description of the datasets is shown in Table 2, and a color map of the land covers is shown in Figure 5.

### 3.2. Performance Metrics

Standard performance metrics for classification tasks were employed to comprehensively evaluate the proposed approach’s performance and compare it with baseline methods. The following metrics were utilized:

Overall Accuracy (OA): Overall accuracy represents the ratio of correctly identified instances to the total number of instances.

(22) $$OA=\frac{1}{N}\sum_{i=1}^{T}CM_{i}$$

where N = total testing sample, T = total diagonal, and CM = confusion matrix.

Average accuracy (AA): Average accuracy is the mean of accuracies obtained for each individual class.

(23) $$AA=\frac{1}{N}\sum_{i=1}^{N}CA_{i}$$

where N is the number of classes and CA_i_ represents class-specific accuracy.

Kappa core (KS): kappa measures the observed agreement between two classifiers compared to the agreement that would be expected purely by chance. This metric can be used to evaluate the reliability and consistency of a classifier on a categorical problem. Kappa is calculated using the following formula:

(24) $$KS=\frac{P_{o}-P_{e}}{1-P_{e}}$$

where P_o_ is the proportion of instances where the two classifiers agree and P_e_ is the proportion of instances where the two classifiers would agree by chance.

### 3.3. Experimental Setup

We experimented on Dell Precision 7920 Workstation, which has the following configuration:

Intel Xeon Gold 5222 3.8 GHz Processor (Intel Corporation, Santa Clara, CA, USA), Kingston 128 GB DDR4 2933 RAM (Kingston Technology Company, Fountain Valley, CA, USA), Kingston 1 TB 7200 RPM SATA HDD (Kingston Technology Company, Fountain Valley, CA, USA), Kingston 500 GB SSD (Kingston Technology Company, Fountain Valley, CA, USA), Nvidia Quadro RTX 4000 8 GB Graphics Card (Nvidia Corporation, Santa Clara, CA, USA), 24 Inch Dell TFT Monitor (Dell, Round Rock, TX, USA), Dell USB Mouse (Dell, Round Rock, TX, USA), Dell KB216 Wired Keyboard (Dell, Round Rock, TX, USA), Microsoft Windows 10 Operating System (Microsoft Corporation, Redmond, WA, USA), Python 3.8 Programming Language (Python Software Foundation (PSF), Wilmington, DE, USA), and Tensor Flow 2.0 open-source Machine Learning Framework (Google, Menlo Park, CA, USA). The Adam optimizer with an initial learning rate of 0.001 accelerates the training process and trains each model for 200 epochs with a batch size of 128.

### 3.4. Comparative Analysis with Baseline Methods

In this subsection, a comprehensive comparative analysis was performed to evaluate the effectiveness of the proposed approach against traditional classification methods and state-of-the-art deep-learning-based methods.

#### 3.4.1. Quantitative Results

We evaluated the performance of several methods under the same experimental environment. For the PU dataset, 95% of the samples were used for training and 5% for validation. The 2DCNN is a five-layer sequential convolutional neural network. It has three convolutional, two max pooling, and one fully connected layer. On the other hand, 3DCNN has three convolutional layers to extract spectral features.

Further, BTA-Net is an attention-based model designed using 1D and 2D convolutional layers to extract spatial features. The HybridSN utilized 2D and 3D CNN layers to improve performance using spatial and spectral features. At the same time, UML applied multiscale depth-wise 1D and 3D convolutional layers for joining spatial and spectral features. SiT and 3DSwinT provide long-range dependency on the spatial and spectral features using ViT to improve the accuracy of the classification of land covers. The performance measures of the proposed CTNet and other methods are shown in Table 3. Table 3 shows that the HybridSN achieved the highest classification accuracy of 97.53% for painted metal sheets, whereas UML classifies Bare Soil with an accuracy of 96.42%. The transformer-based model SiT obtained 96.53% accuracy for the tree class. The proposed model CTNet achieved the highest classification accuracies of 98.65%, 95.37%, 94.17%, 98.76%, and 97.58% for the Asphalt, Gravel, Bitumen, Self-Blocking Bricks, and Shadows classes, respectively.

In the PUC dataset, for nine land covers, 7456 samples are available, which is less than the PU dataset. In addition, to avoid overfitting, we trained all models on 90% samples and validate on 10% samples. Other experimental setups were the same as those used for the PU dataset. The performance for each class and OA, AA, and Kappa value is shown in Table 4. Table 4 shows that the BTA-Net achieved an accuracy of 98.12% for the Self-Blocking Bricks land cover. UML achieved the highest classification, 97.82%, for Asphalt, whereas SiT obtained 98.84% accuracy for Tiles class. The 3DswinT obtained an accuracy of 97.57% for the Meadows class.

The SV dataset contains 54,129 samples. The dataset is divided into 95% and 5% for training and validation. In Table 5, we can see that the 2DCNN performance could be better in several classes. However, 3DCNN improved the performance in the Lettuce_romaine_7wk and Vinyard_vertical_trellis classes. Moreover, BTA-Net has the highest classification accuracy for the Lettuce_romaine_6wk class. The HybridSN discriminates Fallow_rough_plow and Stubble land covers with the highest quantitative value. Further, UML showed improved results in several classes. The SiT methods achieved more than 95% classification accuracy. The 3DSwinT and proposed CTNet achieved similar performance in several classes. However, CTNet dominates in classification accuracy, where there are fewer samples.

The Houston 13 dataset contains very few samples for each class. Therefore, we split the dataset into 90% and 10% for training and validation. The quantitative results of the 2DCNN and 3DCNN are less in several classes, as shown in Table 6. The BTA-Net and HybridSN improve the performance. The UML achieved the highest classification accuracy for the Trees class and 3DSwinT for Non-residential buildings. Moreover, CTNet classification accuracy is highest in the five land covers.

#### 3.4.2. Visual Results Analysis

**Figure 6:**
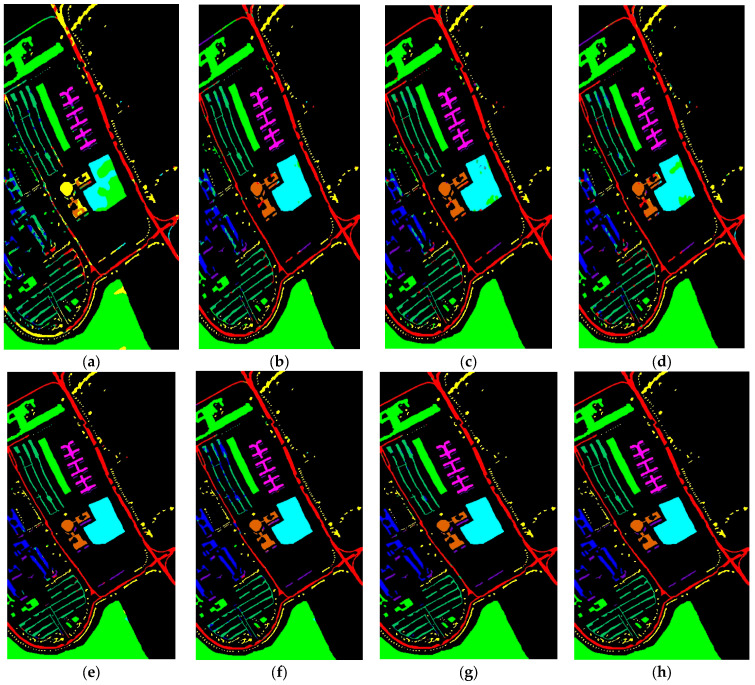
Visual map of land covers using (**a**) 2DCNN (**b**) 3DCNN (**c**) BTA-Net (**d**) HybridSN (**e**) UML (**f**) SiT (**g**) 3DSwinT, and (**h**) CTNet on PU dataset.

**Figure 7:**
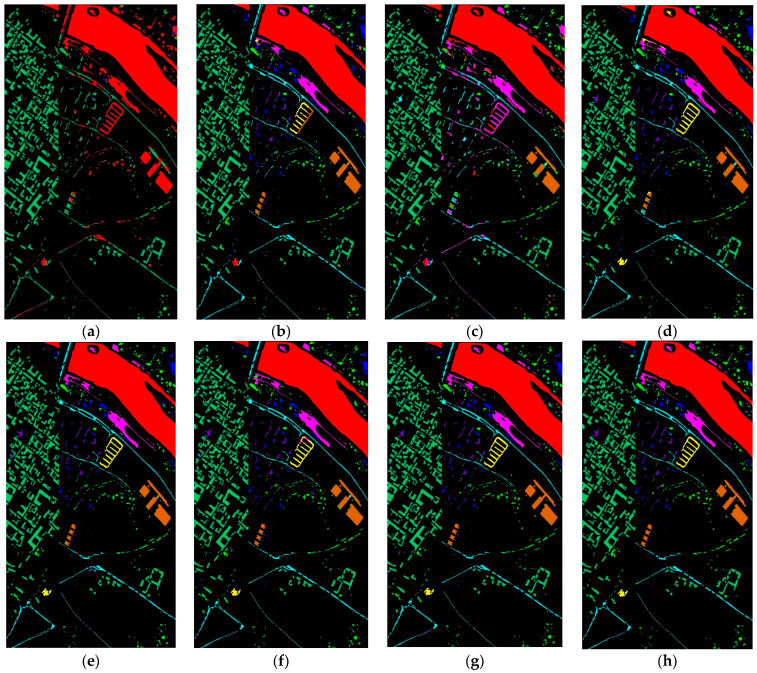
Visual map of land covers using (**a**) 2DCNN (**b**) 3DCNN (**c**) BTA-Net (**d**) HybridSN (**e**) UML (**f**) SiT (**g**) 3DSwinT, and (**h**) CTNet on the PUC dataset.

**Figure 8:**
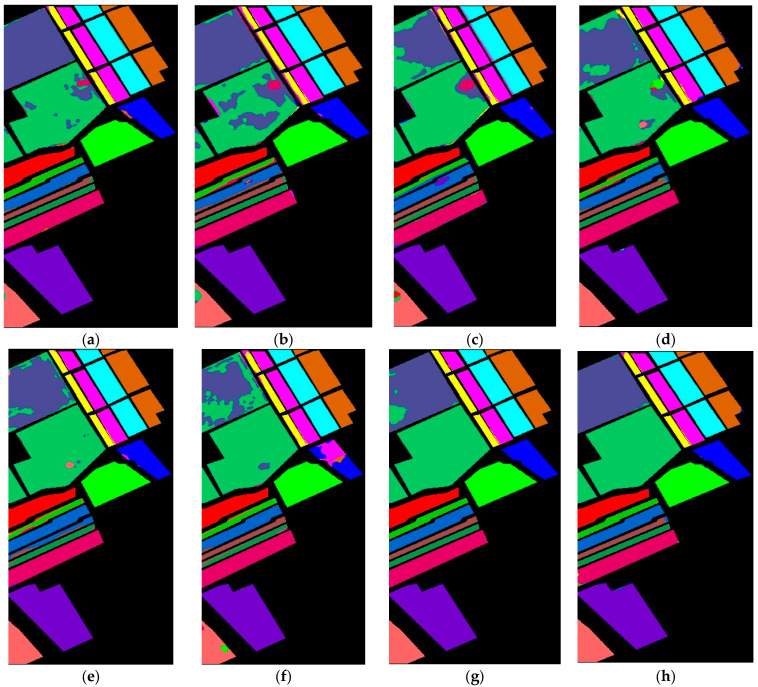
Visual map of land covers using (**a**) 2DCNN (**b**) 3DCNN (**c**) BTA-Net (**d**) HybridSN (**e**) UML (**f**) SiT (**g**) 3DSwinT, and (**h**) CTNet on the SV dataset.

**Figure 9:**
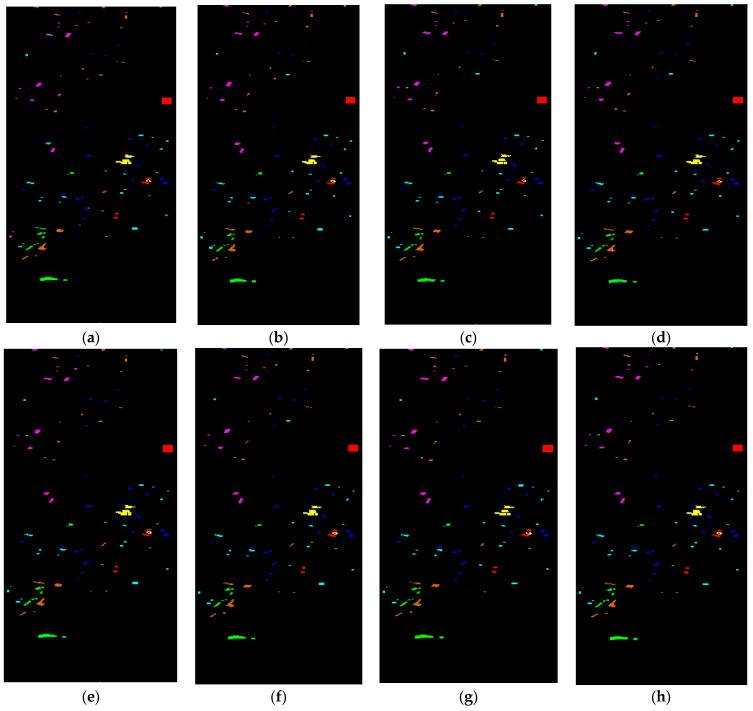
Visual map of land covers using (**a**) 2DCNN (**b**) 3DCNN (**c**) BTA-Net (**d**) HybridSN (**e**) UML (**f**) SiT (**g**) 3DSwinT, and (**h**) CTNet on the Houston13 dataset.

In Figure 6, Figure 7, Figure 8 and Figure 9, we present visual maps for the classes of the PU, PUC, SA, and Houston13 datasets. Specifically, Figure 6 reveals that the 2DCNN-based land cover classification map is not consistently accurate with the ground truth (GT) across various classes. This discrepancy is particularly evident in the Asphalt, Bitumen, Self-Blocking Bricks, and Shadows classes. In contrast, 3DCNN offers enhanced visual maps for several classes. The 3DCNN method displays superior object visualization, mainly producing a map almost identical to the GT for the Painted metal sheets class. The BTA-Net’s representation of the Meadows class outperforms other techniques, while HybridSN’s depiction of the Asphalt class closely aligns with the GT. The UML method leverages global feature attention to refine its land cover classification map, and CTNet’s visualizations closely match the GT in the Trees, Bare Soil, Bitumen, and Shadows classes.

Figure 7 further showcases that the classification maps of 2DCNN, 3DCNN, and BTA-Net for water and Shadows land covers appear noisy. In contrast, HybridSN provides a superior representation for the tiles class. The UML methods provide better visuals in the Asphalts class. Meanwhile, SiT and 3DSwinT improved the visual map of the Tree and Meadows classes. Furthermore, our proposed approach’s classification maps align closely with the GT across multiple classes Water, Trees, Bitumen, Shadows, and Bare Soil.

In Figure 8, the 2DCNN-based method classification map for land covers does not align closely with the GT across various classes. Discrepancies are noticeable in several specific classes. On the other hand, the 3DCNN method offers more accurate visual representations in multiple classes. Compared to the GT, the BTA-Net technique showcases superior object detail, which is especially evident in its near-perfect depiction of the Lettuce_romaine_6wk class. Similarly, HybridSN’s representation of the Fallow_rough_plow and Stubble classes closely mirrors the GT. The UML method refines its portrayal using global feature attention, especially in the Brocoli_green_weeds_2 and Lettuce_romaine_4wk classes. At the same time, SiT showed better visual maps for the Fallow_smooth and Corn_senesced_green_weeds classes. The proposed CTNet improved the visual maps aligning with the GT across Alfalfa, Corn-mintill, Hay-windrowed, Oats, Soybean-clean, Woods, Buildings-Grass-Trees-Drives, and Stone-Steel-Towers classes.

In Figure 9, we can observe that the classification maps from 2DCNN and 3DCNN appear noisy. The BTA-Net offers a refined visualization, particularly for the Non-residential buildings class. The HybridSN excels in representing the Grass healthy class compared to other techniques. UML and 3DSwinT yield superior visualizations for the Trees and Non-residential buildings classes, respectively. Additionally, our suggested approach’s classification maps closely resonate with the GT across various classes.

## 4. Discussion

We evaluated the CTNet on PU, PUC, SV and Houston13 and achieved better quantitative visual results compared to its counterparts 2DCNN, 3DCNN, BTA-Net, HybridSN, UML, SiT, and 3DSwinT, as discussed in Section 3. The proposed model enhances spatial features using virtual RGB and ResNeXt. Further, we enhanced the spectral features using an enhanced attention-based vision transformer (EAVT). ViTs are advanced natural language processing (NLP) techniques representing pairwise interactions among tokens and capturing long-range correlations [35]. The transformer-based technique has been effectively implemented in computer vision applications, and pre-trained transformers now have a robust multipurpose backbone. To implement the classical ViT, we split the input image $I\in R^{H\times W\times D}$ into patches $P\in R^{n\times (p^{H}\times p^{W}\times D)}$ with a size of $p^{H}p^{W}$. The ViT encoder uses alternating multi-head self-attention (MSA) and feed-forward (FF) blocks with layer normalization (LN) to encode and generate embedded data z. The quadratic complexity of the attention mechanism for a given input token is the primary impediment to implementing ViT on high-dimensional data. The complexity of self-attention has been reduced in several research studies, and self-attention has been applied individually instead of pairwise among all tokens to increase the effectiveness of transformers for large numbers of tokens. Our EAVT is inspired by the Swin and convolution self-attention mechanism that enhanced the spectral features.

### 4.1. Patch Size Effect on Model Performance

**Figure 10:**
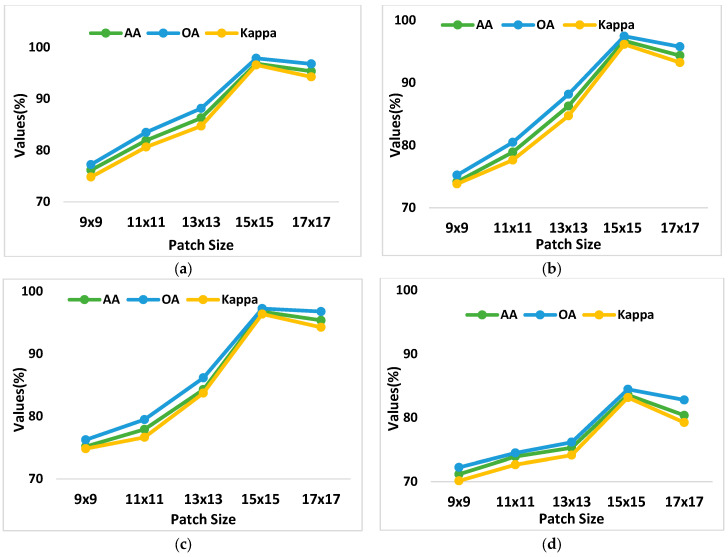
Illustration of the patch size on the OA, AA, and Kappa on PU, PUC, SV, and Houston13 datasets is shown in (**a**), (**b**), (**c**), and (**d**), respectively.

Vision transformers depend on patch size, the length, and the width of the non-overlapping patches created from the input images. The transformer receives tokens that are linearly integrated with these patches. Figure 10 demonstrates that the CTNet performance is lower for 9 × 9 and 11 × 11 patch sizes, while the highest accuracy for classification is attained for 15 × 15 patches. In addition, growing the patch size decreases the accuracy of classification.

### 4.2. Training Loss of the Proposed Model

**Figure 11:**
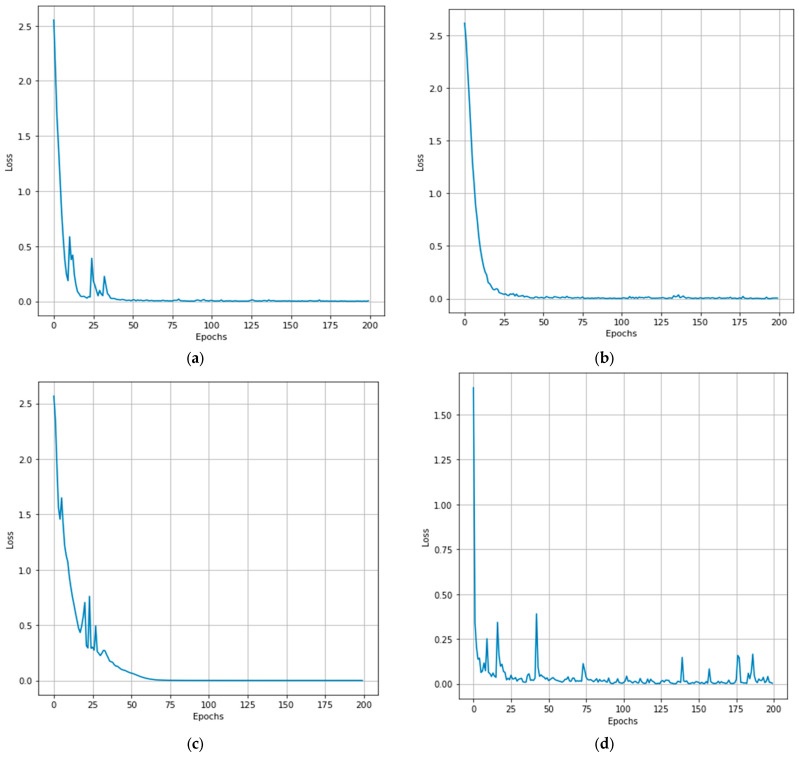
Illustration of the training loss on the PU, PUC, SV, and Houston13 datasets is shown in (**a**), (**b**), (**c**), and (**d**), respectively.

We have calculated the training loss of the CTNet on the four-dataset using the method described in Equation (14) for the PU, PUC, SV, and Houston13 datasets shown in Figure 11. In Figure 11a, the training loss of the proposed method is initially high; after 30 epochs, it reaches zero. On PUC, dataset training loss reaches a value close to zero after 25 epochs. However, the SA dataset reaches a value close to zero after 75 epochs. Furthermore, on the Houston 13 dataset, training loss is relatively high due to the small size of the dataset.

### 4.3. Computation of the Training and Validation Time

In Table 7, we compare the training and validation times of various methods, including 2DCNN [24], 3DCNN [36], BTA-Net [37]. The CNN based require large volume of data for training [38,39]. In addition, we also compared with HybridSN [40], UML [41], SiT [42], 3DSwinT [43], and CTNet. The CTNet demonstrates relatively faster performance than other methods, excluding 2DCNN. This indicates that our approach can reduce computation time and enhance classification efficiency. The high training and validation time for SiT and 3DSwinT are attributed to their deeper network layers, requiring extensive computational cycles per iteration. However, CTNet takes slightly longer than 2DCNN due to utilizing a ResNeXt for spatial feature extraction.

**Figure 12:**
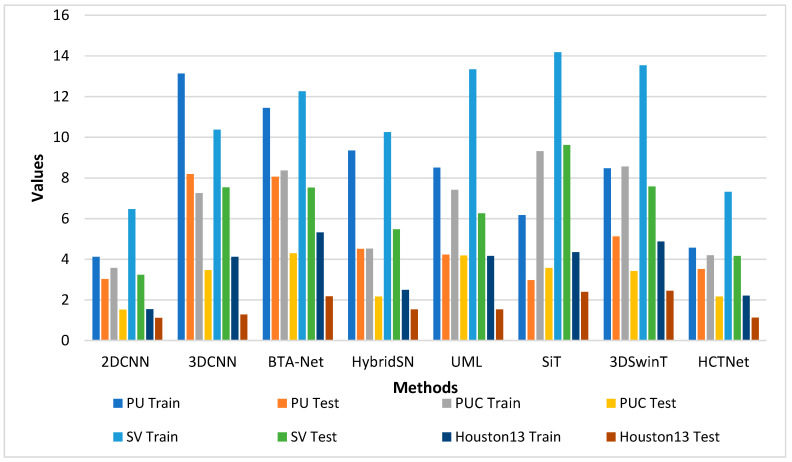
Bar-plot-based comparison of computation time on different datasets.

Further, we plotted the bar plot for the computation time comparison on the PU, PUC, SV, and Houston13 datasets, shown in Figure 12. We can notice that the training time of all the models on the SV training dataset is relatively high. For the Houston13 dataset, the training and test times are the lowest.

### 4.4. Effects of Training Samples (%) on OA Accuracy

**Figure 13:**
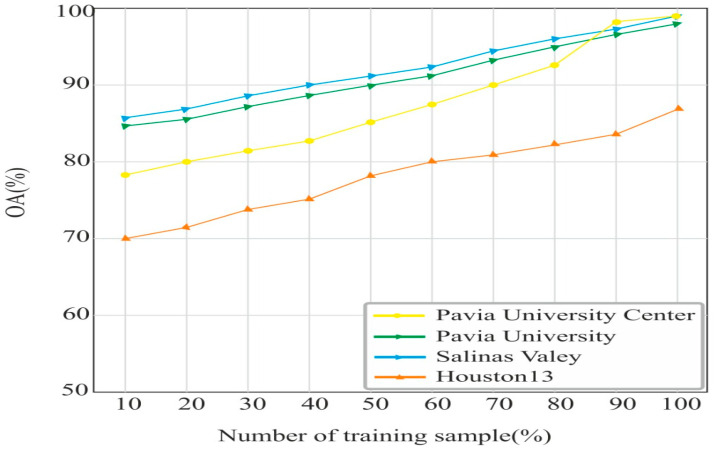
Effect of training sample on OA.

The general thought for the CNN model is that it requires a large volume of training data for better classification performance [44]. We plotted the training sample (in %) and the OA accuracy curve for the PU. The PUC, SV, and Houston datasets are shown in Figure 13. The OA accuracy is less in all the datasets for a small percentage of the training samples. As we increased the samples, the OA accuracy also increased. The highest OA on 90% of the data was obtained in the PUC datasets due to the large samples present in each class of the PUC dataset. The lowest OA accuracy of 84% on 90% training was obtained on the Houston13 dataset due to the fewer samples in each land cover.

### 4.5. Bar Plot Based Comparison

**Figure 14:**
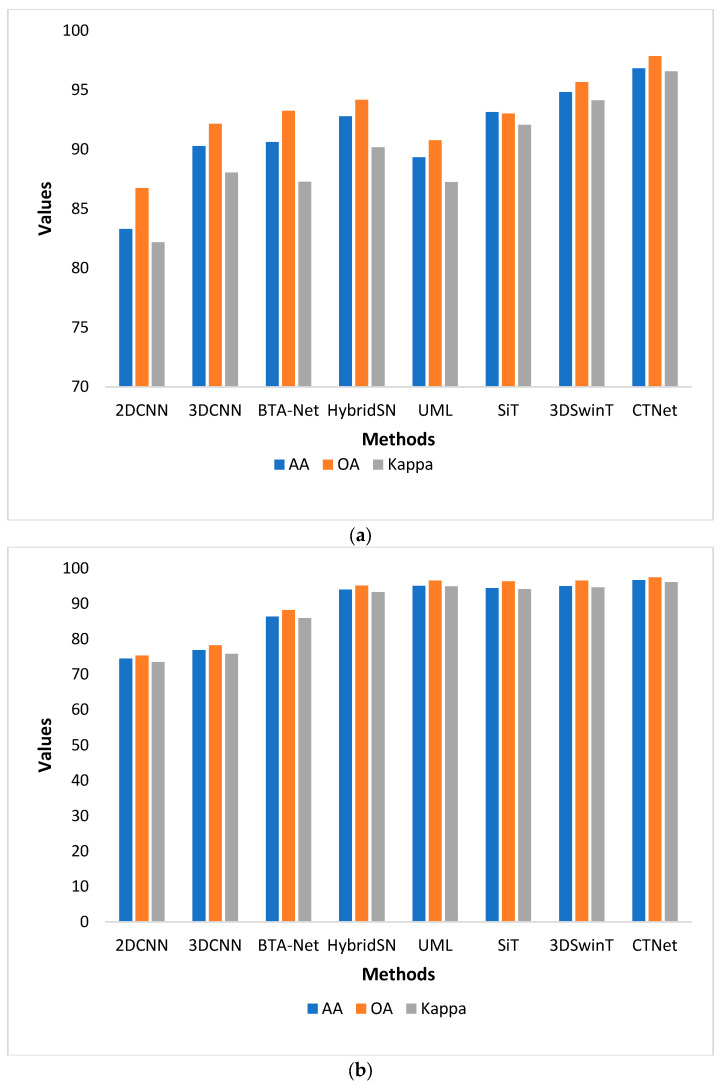

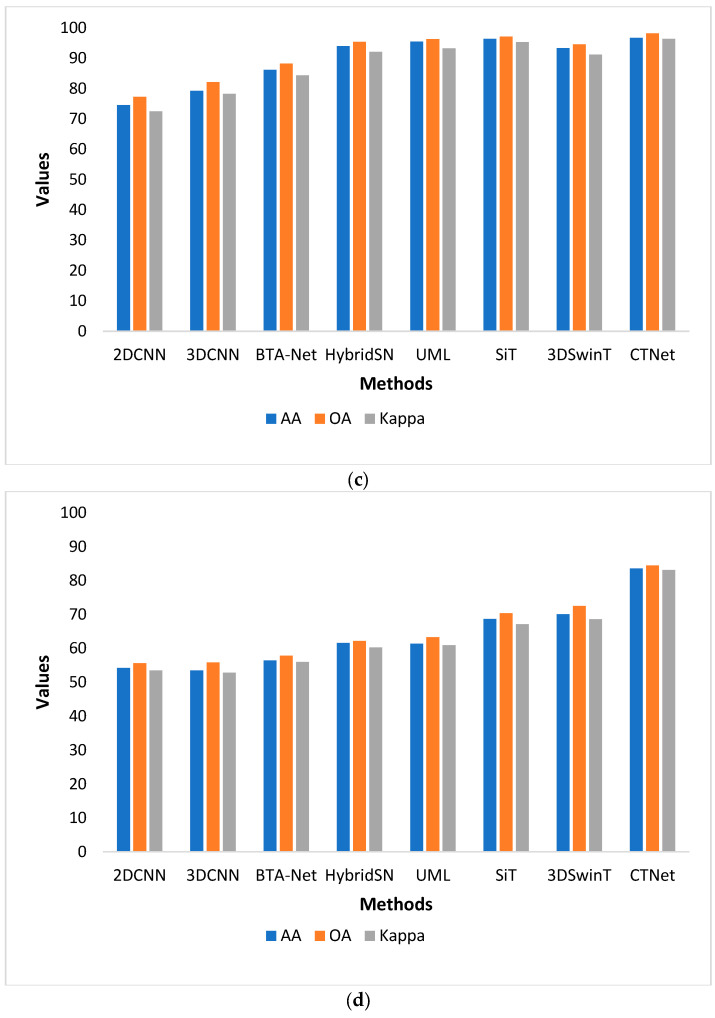
Performance comparison of the different methods on the (**a**) PU, (**b**) PUC, (**c**) SV, and (**d**) Houston13 datasets.

We experimentally evaluated the performance of the 2DCNN [24], 3DCNN [36], BTA-Net [40], HybridSN [37], UML [42], SiT [43], and 3DSwinT datasets [41] and proposed CTNet on the PU, PUC, SV, and Houston13 datasets. All the methods were evaluated under the same experimental conditions for a fair comparison. We plotted these models’ AA, OA, and Kappa scores, as shown in Figure 14. In Figure 14a, we notice that the OA accuracy of classical CNN-based methods is relatively low compared to transformers. The 3DSwinT obtained the second-highest OA of 95.68%, whereas 2DCNN achieved the lowest OA of 86.75% on the PU dataset. On the PUC dataset shown in Figure 14b, UML, SiT, and 3DSwinT, we obtained OA values that were very close to each other. Meanwhile, the proposed CTNet showed superior performance compared to other methods. In Figure 14c, we can observe that the AA accuracy of the SiT is very close to that of the proposed CTNet. At the same time, the lowest AA accuracy can be noticed in the 2DCNN and 3DCNN methods. In addition, the Kappa values of the HybridSN and 3DSWinT are very close to each other. On the Houston 13 dataset shown in Figure 14d, the OA of the classical CNN and transformer-based methods is below 90% due to there being fewer samples in each land cover. The OA of the 2DCNN and 3DCNN are close to each other. Transformer-based methods SiT and 3DSWinT obtained AA values of 68.7% and 70.05%, respectively. Meanwhile, the proposed CTNet achieved an AA value of 83.58%.

## 5. Conclusions

In the proposed study, the fusion of spectral and spatial information has resulted in a remarkable improvement in classification accuracy, surpassing traditional methods and even outperforming deep learning models that do not incorporate RGB data. Integrating RGB and hyperspectral data allows for a more comprehensive characterization of the observed scene, empowering effective discrimination between land cover classes with distinct spectral and spatial patterns. Further, high-dimension spatial features are extracted by pre-trained ResNeXt to improve the spatial features. At the same time, it takes less computation time due to the pre-trained model. In addition, the enhanced attention-based transformer network extracts spectral features to provide a long-range dependency of the features. Furthermore, the fusion of spatial and spectral features enhanced the classification performance. We experimentally evaluated the CTNet on the four standard datasets, PU, PUC, SV, and Houston13. The average accuracies on PU, PUC, SV, and Houston13 is 96.83%, 96.72%, 96.74, and 83.58%, respectively. Moreover, the visual map of the CTNet on these datasets is closer to the GT. The proposed approach can be utilized in agriculture remote sensing to monitor crop health and stress measurement. In addition, it can be also used for the classification of different types of crops. Furthermore, an automated system can be designed for the diagnosis of different types of disease in crops. In the environment of remote sensing, it can be used to monitor the land cover changes, vegetation dynamics, and ecosystem health. In addition, it can be also used for biodiversity assessment by mapping habitats, identifying biodiversity hotspots, and monitoring changes in species distribution.

The major limitations of the proposed method include accurately aligned RGB data with high spatial resolution. Misalignment can disrupt the fusion process and affect classification accuracy. Furthermore, a pre-trained model is required to improve the spatial resolution. The fusion process improved the classification performance, but noise in the data can lead to potential misclassification. Additionally, the approach’s success may be contingent upon the availability of labeled data for training and diverse datasets to achieve optimal performance. We will include axillary data from Radar and other transfer learning and domain adaptation methods in future studies. Further, the interpretability of the hyperspectral image with explainable AI and ensemble learning techniques with real-time applications can be explored.

## Figures and Tables

**Table 1:** Summary of the recent method for HSI classification.

Author	Model	Dataset	OA
Zhang et al. [26]	TMOE-CNN	IP	96.70%
		PU	95.97%
		Houston13	89.36%
Ahmad et al. [27]	WaveFormer	PU	95.66%
		Houston	96.54%
Xu et al. [28]	F-GCN	IP	95.30%
		PU	97.68%
		KSC	99.94%
Shi et al. [29]	AIAF-Defense	PU	95.17%
		Houston 18	71.94%
		SV	96.56%
Ranjan et al. [30]	Siamese network	PU	95.17%
		IP	93.25%
Gao et al. [31]	SSC-SFN	SV	99.48%
		WHU-HI-HanChuan	91.82%
		WHU-HI-HongHu	92.94%
Dang et al. [32]	DCTransformer	IP	94.40%
		Houston	94.89%
Tajasaree et al. [33]	deep-LSTM	PU	93.99%
		IP	99.01%
		KSC	96.72%
Patel et al. [34]	RPDAL	IP	92.78%
		PU	97.85%
		SV	97.94%

**Table 2:** Details of the sample in each land cover with their ground truth and color map.

University of Pavia (PU)				Pavia University Centre (PUC)			
Id	Class	Train	Test	Id	Class	Train	Test
1	Water	742	82	1	Asphalt	6299	332
2	Trees	738	82	2	Meadows	17,717	932
3	Asphalt	735	81	3	Gravel	1994	105
4	Self-Blocking Bricks	727	81	4	Trees	2911	153
5	Bitumen	727	81	5	Painted metal sheets	1278	67
6	Tiles	1134	126	6	Bare Soil	4778	251
7	Shadows	428	48	7	Bitumen	1264	66
8	Meadows	742	82	8	Self-Blocking Bricks	3498	184
9	Bare Soil	738	82	9	Shadows	900	47
Salinas Valley (SV)				Houston13			
Id	Class	Train	Test	Id	Class	Train	Test
1	Brocoli_green_weeds_1	1909	100	1	Grass healthy	311	14
2	Brocoli_green_weeds_2	3540	186	2	Grass stressed	329	36
3	Fallow	1877	99	3	Trees	329	36
4	Fallow_rough_plow	1324	70	4	Water	257	28
5	Fallow_smooth	2544	134	5	Residential buildings	288	31
6	Stubble	3761	198	6	Non-Non-residential buildings	368	40
7	Celery	3400	179	7	Road	399	44
8	Grapes_untrained	10,707	564				
9	Soil_vinyard_develop	5893	310				
10	Corn_senesced_green_weeds	3114	164				
11	Lettuce_romaine_4wk	1015	53				
12	Lettuce_romaine_5wk	1831	96				
13	Lettuce_romaine_6wk	870	46				
14	Lettuce_romaine_7wk	1017	53				
15	Vinyard_untrained	6905	363				
16	Vinyard_vertical_trellis	1717	90				

**Table 3:** Quantitative performance comparison on the PU dataset (in %).

Id.	2DCNN	3DCNN	BTA-Net	HybridSN	UML	SiT	3DSwinT	CTNet
1	85.35	94.17	91.80	95.16	90.53	92.17	94.15	98.65
2	92.18	93.54	92.71	96.47	94.81	96.43	97.63	97.18
3	62.57	81.34	84.05	86.57	85.17	92.62	94.35	95.37
4	91.71	93.18	89.16	91.26	88.62	96.53	95.67	94.94
5	93.87	94.87	95.98	97.53	96.57	94.76	93.75	97.49
6	82.58	91.57	95.36	93.89	96.42	90.85	95.38	95.92
7	80.65	88.94	86.54	84.52	83.53	91.74	92.14	94.17
8	78.64	90.67	87.28	96.76	80.27	91.17	96.81	98.76
9	82.16	83.67	92.79	92.89	89.15	92.04	91.59	97.58
AA	83.30	90.27	90.63	92.78	89.34	93.15	94.83	96.83
OA	86.75	92.15	93.24	94.17	90.76	93.03	95.68	97.87
Kappa	82.17	88.05	87.26	90.18	87.25	92.07	94.13	96.58

**Table 4:** Quantitative performance comparison on the PUC Dataset (in %).

Id.	2DCNN	3DCNN	BTA-Net	HybridSN	UML	SiT	3DSwinT	CTNet
1	56.38	55.34	64.52	88.28	86.62	88.92	89.25	96.72
2	77.53	82.28	82.38	87.58	93.32	94.65	92.53	95.48
3	84.18	87.68	92.32	96.74	97.82	96.16	94.46	95.19
4	75.32	72.27	98.12	97.36	96.32	96.25	96.34	96.87
5	81.96	84.54	87.54	96.32	96.92	97.98	94.54	98.57
6	90.28	93.26	96.87	98.66	99.25	98.84	97.73	96.45
7	46.25	65.78	78.42	92.26	94.87	95.28	97.64	97.82
8	82.78	86.14	90.63	94.86	95.74	96.26	97.57	97.14
9	74.92	62.89	96.18	93.68	96.85	94.45	95.25	96.27
AA	74.44	76.89	86.35	94.01	95.10	94.42	95.03	96.72
OA	75.34	78.24	88.23	95.17	96.58	96.36	96.54	97.46
Kappa	73.48	75.84	85.98	93.28	94.92	94.13	94.62	96.16

**Table 5:** Quantitative performance comparison on the SV Dataset (in %).

Id.	2DCNN	3DCNN	BTA-Net	HybridSN	UML	SiT	3DSwinT	CTNet
1	87.52	64.25	85.27	84.32	89.25	96.72	95.36	97.85
2	78.63	88.76	85.64	85.48	97.53	95.42	93.42	96.15
3	77.81	91.24	90.52	96.37	94.46	95.84	89.25	98.24
4	65.27	72.28	80.62	98.56	96.34	98.38	88.12	96.52
5	87.78	88.67	93.25	95.62	94.54	97.56	95.78	97.21
6	68.46	67.89	75.48	98.46	97.73	97.94	96.92	95.54
7	58.35	51.48	71.34	97.82	97.64	98.52	98.64	97.12
8	65.28	66.78	74.94	95.25	98.28	97.38	92.36	98.67
9	58.92	64.96	88.36	88.28	86.62	88.92	84.52	92.25
10	68.84	76.43	68.74	87.58	93.32	96.65	92.38	94.52
11	78.65	88.75	91.82	96.74	97.82	96.16	94.32	96.15
12	81.52	76.38	94.76	97.36	96.32	96.25	97.12	98.52
13	82.42	95.57	98.15	96.32	98.12	97.98	95.54	98.17
14	85.57	94.32	95.21	95.66	97.25	96.14	96.87	98.86
15	72.64	85.65	90.18	92.26	94.87	95.28	88.42	96.24
16	75.85	94.36	96.34	97.82	97.13	97.48	93.87	97.94
AA	74.59	79.24	86.19	93.99	95.50	96.41	93.31	96.74
OA	77.28	82.16	88.27	95.36	96.35	97.17	94.54	98.25
Kappa	72.45	78.22	84.35	92.15	93.26	95.28	91.19	96.37

**Table 6:** Quantitative performance comparison on Houston13 Dataset (in %).

Id.	2DCNN	3DCNN	BTA-Net	HybridSN	UML	SiT	3DSwinT	CTNet
1	44.53	47.37	56.38	67.82	72.43	65.28	66.78	74.94
2	52.67	68.72	46.86	57.28	54.85	58.92	64.96	88.36
3	48.14	49.85	41.58	45.84	73.76	48.84	46.43	68.74
4	61.34	46.86	58.93	63.76	68.78	78.65	88.75	91.82
5	54.53	65.67	66.37	62.94	72.13	81.52	76.38	94.76
6	72.72	42.92	68.92	65.87	75.52	82.42	95.57	95.10
7	45.48	52.94	56.38	67.82	42.32	65.28	51.48	71.34
AA	54.20	53.47	56.48	61.62	61.40	68.70	70.05	83.58
OA	55.64	55.84	57.83	62.17	63.28	70.36	72.54	84.46
Kappa	53.48	52.84	55.98	60.28	60.92	67.13	68.62	83.16

**Table 7:** Comparison of training and validation times of various methods.

Methods	PU		PUC		SV		Houston13	
	Train (s)	Test (s)	Train (s)	Test (s)	Train (s)	Test (s)	Train (s)	Test (s)
2DCNN [24]	247.2	3.02	214.2	1.52	387.6	3.23	92.4	1.12
3DCNN [36]	788.4	8.19	435	3.46	622.2	7.54	247.2	1.28
HybridSN [37]	561	4.51	271.2	2.17	615.6	5.47	149.4	1.53
BTA-Net [40]	687	8.06	502.2	4.29	735.6	7.52	319.2	2.18
3DSwinT [41]	508.2	5.12	513.6	3.42	812.4	7.58	292.2	2.45
UML [42]	510.6	4.23	445.2	4.18	800.4	6.26	249.6	1.53
SiT [43]	370.2	2.97	559.2	3.57	850.8	9.62	261	2.39
CTNet	273.6	3.52	251.4	2.16	439.2	4.16	132.6	1.13

## Data Availability

Dataset used in the study can be downloaded from http://lesun.weebly.com/hyperspectral-data-set.html (accessed on 14 October 2023).

## Abbreviations

HSI	Hyperspectral imaging
CTNet	Convolutional transformer network
RGB	Red blue green
PCA	Principal component analysis
EAVT	Enhanced attention-based vision transformer
AA	Average accuracy
OA	Overall accuracy
PU	Pavia university
PUC	Pavia university Centre scene
SV	Salina velley
ML	Machine learning
DL	Deep learning
CNN	Convolution neural network
SVM	Support vector machine
MNF	Minimum noise fraction
NMF	Non-negative matrix factorization
BN	Batch normalization
ReLU	Rectified linear unit
FCN	Fully convolution network
ROSIS	Reflective Optics System Imaging Spectrometer
CM	Confusion matrix
GT	Ground truth
NLP	Natural language processing
ViT	Vision transformer
AI	Artificial intelligence
TMOE-CNN	Tree-shaped multi objective evolutionary CN
F-GCN	Fuzzy graph convolutional network
KSC	Kennedy space Centre
AIAF-Defense	Attack-invariant attention feature-based defense
DCTransformer	Discrete cosine transform
RPDAL	Reinforced pool-based deep active learning
